# The Effect of Clavicular Tunnel Position on Reduction Loss in Patients with Acute Acromioclavicular Joint Dislocations Operated with a Single‐Bundle Suspensory Loop Device

**DOI:** 10.1111/os.14095

**Published:** 2024-05-20

**Authors:** Ahmet Senel, Murat Eren, Omer Cihan Batur, Oguz Kaya, Selman Sert, Sefa Key

**Affiliations:** ^1^ Orthopedics and Traumatology Department Istanbul Training and Research Hospital Istanbul Turkey; ^2^ Orthopedics and Traumatology Department Elazig Fethi Sekin City Hospital Elazig Turkey; ^3^ Department of Orthopedics and Traumatology Firat University Faculty of Medicine Elazig Turkey

**Keywords:** Acromioclavicular Joint Dislocation, Clavicular Tunnel Position, Reduction Loss, Single Bundle, Suspensory Loop Device

## Abstract

**Objective:**

The treatment of acromioclavicular joint (ACJ) dislocations offers numerous options, and ongoing debates persist regarding their comparative effectiveness. Among these options, the suspensory loop device (SLD) is one of the most favored treatment modalities. Despite the observed high reduction loss rate associated with SLD, the treatment yields favorable clinical outcomes. This study aimed to investigate the clinical outcomes of patients with acute type 3 and 5 ACJ dislocations who underwent open and arthroscopic procedures using a single‐bundle SLD, and to evaluate the effect of clavicular tunnel position on reduction loss.

**Methods:**

Thirty‐seven eligible patients diagnosed with acute type 3 and type 5 ACJ dislocation who underwent open and arthroscopic surgery with a single‐bundle SLD between January 2015 and March 2022 were evaluated retrospectively. Demographic data and radiological measurements including coracoclavicular (CC) interval, clavicle length (CL), and implant distance (ID) were recorded. The ID/CL ratio was calculated and a value between 0.17 and 0.24 was considered as “acceptable implant position”. Reduction loss and other complications were noted. Patients were divided into two groups: open (Group 1) and arthroscopic (Group 2). Constant Murray Score (CMS) and Visual Analog Scale (VAS) were used for clinical and functional outcomes. Non‐parametric tests were used for statistical analysis of variables.

**Results:**

The study included six females (16.2%) and 31 males (83.8%) with a mean age of 40.2 ± 14.7 years (range: 20–75). The mean follow‐up period was 22.3 ± 16.7 months (range: 6–72). The average time from trauma to surgery was 6.3 ± 5.3 days (range: 1–18). At the last follow‐up, the CMS was 89.3 ± 8.8 and the VAS score was 2.1 ± 0.9. The mean ID/CL ratio was 0.19 ± 0.1 and 19 patients (51.4%) were between 0.17 and 0.24. Reduction loss was observed in nine patients (24.3%). There were no significant differences between Group 1 and Group 2 regarding operation time (*p* = 0.998), ID/CL ratio (*p* = 0.442), reduction loss (*p* = 0.458), CMS (*p* = 0.325), and VAS score (*p* = 0.699). Of the 28 patients without reduction loss, 16 had an ID/CL ratio between 0.17 and 0.24 (*p* = 0.43). Furthermore, within the 0.17–0.24 interval, CMS was higher with an average of 91.8 ± 5.1 compared to the other intervals (*p* = 0.559).

**Conclusion:**

The clinical and functional outcomes of acute type 3 and type 5 ACJ dislocation operated open and arthroscopically with single‐bundle SLD are similar and satisfactory. A clavicular tunnel position in the range of 0.17–0.24 (ID/CL ratio) is recommended to maintain postoperative reduction.

## Graphical abstract

In this study, the effect of the clavicular tunnel distance/clavicular length ratio on the reduction loss in AC joint dislocation operated with single bundle SLD was evaluated. Based on our results and previous anatomical studies, we think that position of the clavicular tunnel with a ratio between 0.17 and 0.24 will maintance the reduction.

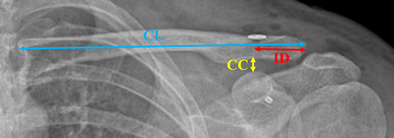

## Introduction

Acromioclavicular joint (ACJ) dislocation accounts for 11% of shoulder girdle traumas. The incidence ranges from 2 to 4.5 per 10,000 person‐years, and it is commonly observed in males during the second and third decades of life. Although it is most commonly occurring after sports injuries, traffic accidents and falls are other trauma mechanisms.^1^, ^2^ Treatment is decided according to the classification described by Rockwood, which is divided into six subgroups according to the displacement rate and direction of the ACJ.^3^ According to this classification, type 1 and 2 injuries are treated conservatively, whereas surgical treatment is preferred in high‐grade type 4, 5, and 6 injuries.^4^ Treatment of type 3 injuries is controversial and should be decided according to patient expectations, injury, and evaluation of response to conservative treatment.^5^, ^6^

The aim of surgical treatment of ACJ dislocations is to achieve joint reduction and subsequently maintain stabilization. Many treatment options such as K‐wire, hook plate, biological (graft) reconstruction, screw fixation, and suture anchor have been described to achieve joint reduction. However, each treatment option has its own set of advantages and disadvantages, and there is no consensus on the superiority of one treatment over another.^7^ Some implant types used in treatment act as temporary support by maintaining ACJ reduction until ligament healing is completed. Recently, satisfactory clinical outcome and pain relief have been reported with the suspensory loop device (SLD), which can be performed open and arthroscopically for this purpose.^8^ Although previous studies have reported reduction loss and other complications associated with the device or surgical technique in treatments with SLD, these have been shown not to affect clinical results.^9^

In the SLD surgical technique, the clavicular tunnel is prepared by drilling approximately 25–40 mm medial to the ACJ.^10^, ^11^ In this way, the tunnel is positioned in the area where the coracoclavicular (CC) ligaments (trapezoid and conoid ligaments) at the inferior edge of the clavicle are attached. Cadaveric studies have shown that the length of the clavicle and the distance from the lateral edge of the clavicle to attachment points of the CC ligaments vary individually, but the ratio of this distance to the total length of the clavicle is constant. This ratio is 0.17 for the trapezoid ligament and 0.24 for the conoid ligament.^12^

The aim of this study is to investigate the clinical and radiological outcomes in acute type 3 and 5 ACJ dislocations operated through open and arthroscopic procedures with a single‐bundle SLD and to assess the impact of clavicular tunnel position on the reduction loss.

## Methods

**FIGURE 1:**
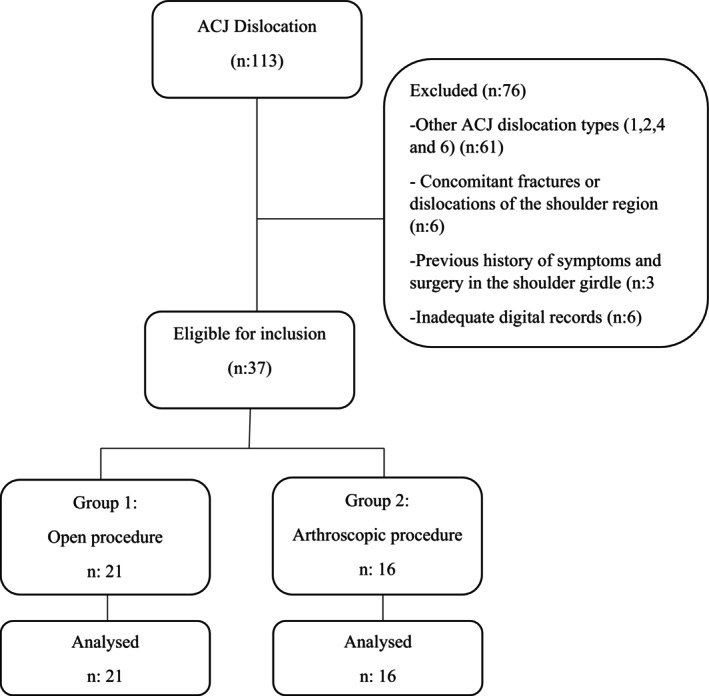
Flow chart of the study.

This retrospective cohort study was approved by our institutional ethics committee (date: 04.08.2023 no: 202). Patients who operated with a diagnosis of ACJ dislocation between January 2015 and March 2022 were determined from the digital hospital archive. The inclusion criteria for this study were as follows: (i) patients aged 18 years and older; (ii) diagnosed with isolated, acute (<3 weeks), Rockwood classification type 3 and type 5 ACJ dislocation; (iii) treated with a single‐bundle SLD; (iv) provided written informed consent; and (v) had at least 6 months of follow‐up. The exclusion criteria comprised: (i) additional concurrent fractures or dislocations of the shoulder region (coracoid, acromion, clavicle, and humerus fracture or glenohumeral dislocation); (ii) a history of symptoms and surgery in the shoulder girdle (shoulder instability, rotator cuff, and biceps tendon disorders); and (iii) inadequate digital records. Finally, 37 patients who met the criteria were included in the study (Figure 1). Patients were divided into Group 1 for open surgery and Group 2 for arthroscopic surgery.

### Clinical and Radiological Evaluation

**FIGURE 2:**
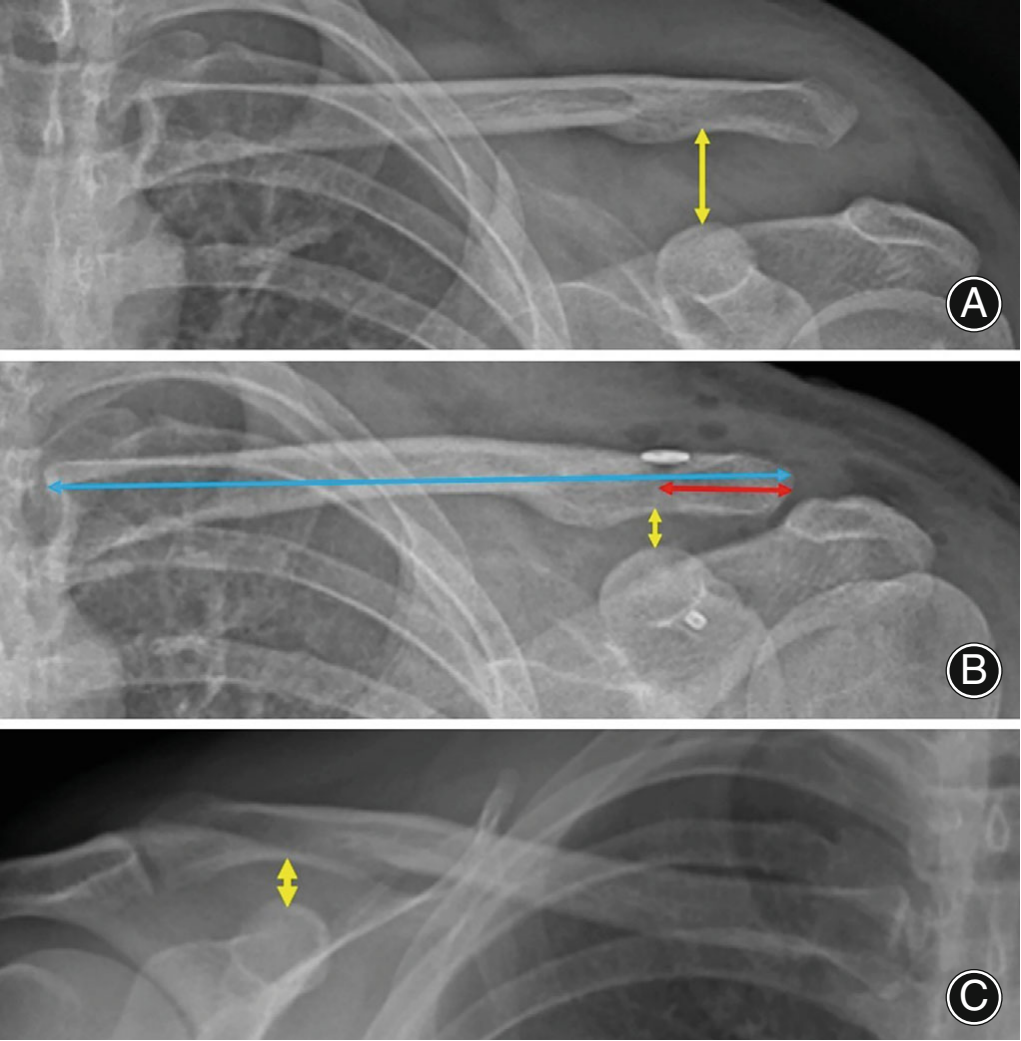
Illustration of all radiological measurements. The coracoclavicular (CC) interval is shown with a yellow double‐headed arrow, the implant distance (ID) with a red double‐headed arrow, and the clavicle length (CL) with a blue double‐headed arrow. a. Preoperative CC interval on the affected side, b. Postoperative CC interval, ID, CL on the affected side and c. CC interval on the unaffected side.

Age, gender, side, mechanism of injury, type of ACJ dislocation, type of surgery (open or arthroscopic), accompanying injuries, time to surgery, surgery duration and complications were documented. Clinical and functional scores were assessed using Constant Murray Score (CMS)^13^ and Visual Analog Scale (VAS). Bilateral anteroposterior shoulder radiographs at preoperative, postoperative day 1, and last follow‐up were used for radiological evaluation. ACJ reduction was assessed by coracoclavicular (CC) interval, which is the vertical distance between the superior edge of the coracoid and the inferior edge of the clavicle. The CC interval was calculated preoperatively, on postoperative day 1, and at the last follow‐up on the operated and unaffected side using a digital caliper on picture archiving communication systems (Figure 2). An increase of more than 50% in the CC interval of the operated side at the last follow‐up compared to postoperative day 1 was considered as “reduction loss”. Additionally, two other values were measured to investigate the effect of implant position on reduction loss. The first is the implant distance (ID), representing the distance between the lateral edge of the clavicle and the clavicular tunnel. The second is clavicle length (CL), defined as the distance between the lateral and medial edges of the clavicle on the operated side (Figure 1). Finally, the ID/CL ratio was calculated. A ratio between 0.17 and 0.24 was considered as a reference for “acceptable implant position”.^12^ All measurements were made by two senior surgeons who were blinded to the demographic and group information of patients. In case of disagreement between examiners, consensus was reached by calculating the arithmetic average of both measurements.

### Surgical Techniques

All patients diagnosed with type 5 ACJ dislocation who were suitable for surgery were operated on. Surgery was preferred for patients diagnosed with type 3, particularly for individuals who actively use their upper extremities and elite athletes, to expedite their return to activity by achieving early pain control and rehabilitation. Moreover, patients with thin soft tissues that may lead to skin problems later and those with cosmetic concerns were indicated for surgery.

All patients were operated on under general anesthesia and in the beach chair position. The upper limb was sterilely prepped and draped, and appropriate antibiotic prophylaxis was administered prior to incision. The decision of open or arthroscopic approach was made by the surgeon.

### Open Surgery

A 5–6 cm vertical skin incision was made starting 3–4 cm medial to the ACJ towards the coracoid. The deltopectoral fascia was incised and the coracoid process was visualized. The anteroposterior edges of the clavicle were determined approximately 35 mm medial to the ACJ, and a 2.4 mm guide pin was placed from the midpoint in the superior–inferior direction. The clavicular tunnel was prepared with a 4 mm cannulated drill over the guide pin. Then, the medial and lateral edges of the coracoid base were determined and the coracoid tunnel prepared in the same way. A nitinol suture passer wire was sent from the clavicular tunnel to the coracoid tunnel. Ethibonds attached to one edge of the SLD (Asfix, Onarge, TURKEY) were passed through the tunnels using nitinol wire. The nitinol wire was removed. The ethibonds were pulled until the endobutton of the SLD, which would be placed under the corocoid, passed the coracoid tunnel. Then, the ACJ was reduced under fluoroscopic guidance. The other endobutton of the SLD, which was located on the upper edge of the clavicle, was fixed by tying a knot to maintain the reduction. After the reduction was checked again, the wounds were closed.

### Arthroscopic Surgery

Firstly, the glenohumeral joint was examined with a 30° scope through standard posterior and anterior portals. Then, the rotator interval was opened with a radiofrequency device to reach the coracoid process. At this stage, the scope was changed to a 70° scope to visualize the lateral, superior, and inferior sides of the coracoid base. Afterwards, the clavicle was incised as described for the open procedure. The drill guide was inserted through the anterior portal and positioned on the inferior surface of the coracoid. A 2.4 mm guide pin was then inserted through the drill sleeve from the clavicle to the coracoid. The drill sleeve was removed and a 4 mm cannulated drill was used to prepare the clavicle and coracoid tunnels through the guide pin. The guide pin was removed and the cannulated drill was left in the tunnels. The nitinol wire was passed through the cannulated drill until it was visible on the inferior edge of the coracoid. A grasper was utilized to remove the nitinol wire through the anterior portal. The cannulated drill was then removed. In the following stages, SLD transport, joint reduction, and knotting of the implant were performed as described in the open procedure. After fluoroscopic control, the portal and incisions were sutured.

No implant, such as K‐wire or screw, was placed in the ACJ in either the open or arthroscopic technique due to the risk of joint degeneration and implant‐related complications. Primary ligament repair was not performed for CC ligaments.

### Postoperative Course

Sling immobilizer was used in all patients for 4 weeks postoperatively. Passive elbow motion was performed on postoperative day 1 and Codman's exercises were initiated in postoperative week 2. Strengthening exercises and physiotherapy tailored to the patient's condition commenced in the sixth postoperative week. Gradual weight lifting was permitted starting from the second month postoperatively. Depending on the patient's follow‐up, the appropriate patients returned to work in the second to third month postoperatively. Contact sports were not allowed until 6–8 months postoperatively.

### Statistical Analysis

SPSS software version 25.0 for Windows (IBM Corp., New York, USA) was used for statistical analyses. Descriptive statistics were expressed as mean, standard deviation, median, minimum and maximum values for numerical variables, and as numbers and percentages for categorical variables. The Kolmogorov–Smirnov test was used to test distributions, and non‐parametric tests were performed. Numerical variables between two independent groups were analyzed using the Mann–Whitney U test, and comparisons among three or more independent groups were conducted using the Kruskal–Wallis test. The Wilcoxon test was utilized to assess the relationship between two dependent groups. Repeated numerical variables were tested using Friedman's analysis. Fisher's exact test was used to analyze independent two categorical data. Chi‐square test was used to analyze multiple categorical data. The Intraclass Correlation Coefficient (ICC) was used to assess inter‐observer agreement for radiological measurements. Two‐way random mode was used to calculate the ICC with a 95% confidence interval. The ICC was interpreted as follows: below 0.50: poor, between 0.50 and 0.75: fair, between 0.75 and 0.90: good, above 0.90: excellent. *p* < 0.05 was considered statistically significant.

## Results

### Demographic Features of Patients

Of the 37 patients included in the study, six (16.2%) were female and 31 (83.8%) were male. The mean age was 40.2 ± 14.7 years (range: 20–75) and the mean follow‐up period was 22.3 ± 16.7 months (range: 6–72). According to the Rockwood classification, type 3 ACJ dislocation was diagnosed in 23 patients (62.2%) and type 5 ACJ dislocation in 14 patients (37.8%). Twenty‐six patients (70.3%) had a history of falls, four patients (10.8%) had a history of sports injuries, and seven patients (18.9%) had a history of vehicle accidents (motor, car, etc.). Thirty‐five patients (94.6%) had no concomitant trauma, while one patient (2.7%) had a head trauma, and one patient (2.7%) had an ankle ligament injury. The mean time from trauma to surgery was 6.3 ± 5.3 (range: 1–18) days (Table 1).

**TABLE 1:** Demographic features of patients.

Parameters	Total	Open	Arthroscopic	Statistic value	*p*
	n:37 (100%)	n:21 (56.8%)	n:16 (43.2%)		
Gender (n)					
Female	6 (16.2%)	5 (23.8%)	1 (6.3%)	2.061	0.206^b^
Male	31 (83.8%)	16 (76.2%)	15 (93.8%)		
Age (years)^a^	40.2 ± 14.7 [37]	42.7 ± 16.8 [45]	37.1 ± 11.3 [36]	−0.921	0.357^c^
Trauma type (n)					
Falling	26 (70.3%)	15 (71.4%)	11 (68.8%)	0.252	0.615^d^
Sport Injury	4 (10.8%)	3 (14.3%)	1 (6.3%)		
Vehicle Accident	7 (18.9%)	3 (14.3%)	1 (6.3%)		
Side (n)					
Right	18 (48.6%)	9 (42.9%)	9 (56.3%)	0.652	0.515^b^
Left	19 (51.4%)	12 (57.1%)	7 (43.7%)		
Additional trauma (n)					
None	35 (94.6%)	21 (100%)	14 (87.4%)	3.504	0.173^d^
Head trauma	1 (2.7%)	0 (0.0%)	1 (6.3%)		
Ankle ligament injury	1 (2.7%)	0 (0.0%)	1 (6.3%)		
ACJ dislocation type (n)					
3	23 (62.2%)	13 (61.9%)	10 (62.5%)	0.001	>0.999^b^
5	14 (37.8%)	8 (38.1%)	6 (37.5%)		
Time from trauma to surgery (days)^a^	6.3 ± 5.3 [4]	5.2 ± 5.7 [3]	7.8 ± 5.3 [6.5]	−1.728	0.084^c^
Operation time (minutes)^a^	86.9 ± 23.6 [80]	85.7 ± 20.1 [80]	88.6 ± 28.1 [87.5]	−0.015	0.998^c^
Follow‐up time (months)^a^	22.3 ± 16.7 [14]	25.7 ± 15.8 [24]	17.8 ± 17.4 [12]	−1.862	0.063^c^

Abbreviation: ACJ, acromioclavicular joint.

a Data are presented as means ± standard deviations [median].

^b^ Fisher's Exact.

^c^ Mann–Whitney U.

^d^ Chi‐square.

Twenty‐one patients (56.8%) underwent open surgery (Group 1), while 16 patients (43.2%) underwent arthroscopic surgery (Group 2). The mean age for Group 1 was 42.7 ± 16.8 years (median: 45, range: 20–75), and for Group 2, it was 37.1 ± 11.3 years (median: 36, range: 21–56). The mean operation time was 85.7 ± 20.1 minutes (median: 80) (range: 60–130) in Group 1 and 88.6 ± 28.1 minutes (median: 87.5) (range: 54–140) in Group 2 (*p* = 0.998) (Table 1).

### Radiological Evaluation

According to radiological measurements, the mean CL was 152.6 ± 15.0 millimeters (mm) (median: 150.0) (range: 130.0–180.7) in Group 1 and 159.6 ± 17.0 mm (median: 159.9) (range: 129.9–187.8) in Group 2 (*p* = 0.101). The mean ID was 30.2 ± 10.0 mm (median: 33) (range: 7.1–50.0) and 29.5 ± 10.3 mm (median: 30.1) (range: 9.1–47.9), respectively. Accordingly, the mean ID/CL ratio was 0.20 ± 0.7 (median: 0.22) (range: 0.05–0.28) in Group 1 and 0.19 ± 0.1 (median: 0.19) (range: 0.06–0.28) in Group 2. There was no statistical difference between the groups for both ID and ID/CL ratio (*p* = 0.69 and *p* = 0.442, respectively). The CC distance measured preoperatively, on postoperative day 1 and at the last follow‐up was statistically significant in both groups (*p* < 0.01 for both groups). The mean percentage change of the difference between last follow‐up and postoperative day 1 was 31.6 ± 25.3 (median: 33.3) (range: −22.6‐75.0) in Group 1 and 44.9 ± 36.2 (median: 41.2) (range: 0–117.5) in Group 2 (*p* = 0.358). Reduction loss was noted in four patients (19.0%) in Group 1 and five patients (31.3%) in Group 2 (*p* = 0.458) (Table 2). All radiological measurements showed significant inter‐observer correlations (Table 3).

**TABLE 2:** Comparison of radiological measurements.

Parameters	Total	Open	Arthroscopic	Statistic value	*p*
	n:37 (100%)	n:21 (56.8%)	n:16 (43.2%)		
Distance from lateral edge of clavicle to clavicular tunnel (ID) (mm)^a^	29.9 ± 10.0 [31.1]	30.2 ± 10.0 [33]	29.5 ± 10.3 [30.1]	−0.399	0.69^c^
Clavicle length (CL) (mm)^a^	155.7 ± 16.0 [154.6]	152.6 ± 15.0 [150]	159.6 ± 17.0 [159.9]	−1.641	0.101^c^
ID/CL ratio^a^	0.19 ± 0.1 [0.20]	0.20 ± 0.7 [0.22]	0.19 ± 0.1 [0.19]	−0.769	0.442^c^
CC distance (mm)^a^					
Preoperative	17.7 ± 4.8 [17.7]	17.3 ± 4.5 [16.0]	18.3 ± 5.3 [19.2]	−0.797	0.425^c^
Postoperative day 1	8.4 ± 2.9 [8.5]	8.3 ± 3.1 [8.5]	8.6 ± 2.7 [8.2]	−0.537	0.591^c^
Last follow‐up	11.3 ± 3.9 [10.7]	10.6 ± 3.7 [10.2]	12.2 ± 4.1 [11.1]	−0.951	0.342^c^
*p* ^**d**^	<0.01	<0.01	<0.01		
Change between postoperative day and last follow‐up (%)	37.3 ± 30.8 [37.2]	31.6 ± 25.3 [33.3]	44.9 ± 36.2 [41.2]	−0.920	0.358^c^
Unaffected side	8.5 ± 2.1 [8.7]	7.9 ± 2.1 [8.1]	9.2 ± 1.9 [9.1]	−1.504	0.133^c^
Reduction loss (n)					
None	28 (75.7%)	17 (80.9%)	11 (68.7%)	0.735	0.458^b^
Yes	9 (24.3%)	4 (19.1%)	5 (31.3%)		

Abbreviations: CC, Coracoclavicular; CL, Clavicle length; ID, Implant distance.

^a^ Data are presented as means ± standard deviations [median].

^b^ Fisher's Exact.

^c^ Mann–Whitney U.

^d^ Friedman.

**TABLE 3:** Intraclass correlation coefficient for inter‐observer radiological measurement.

Parameters	ICC	%95 Confidence Interval	*p*
ID	0.989	0.979–0.994	<0.01
CL	0.996	0.992–0.998	<0.01
Preoperative CC distance	0.952	0.907–0.975	<0.01
Postoperative 1st day CC distance	0.933	0.870–0.965	<0.01
Final follow‐up CC distance	0.930	0.863–0.964	<0.01
Unaffected Side CC distance	0.940	0.884–0.969	<0.01

Abbreviations: CC, Coracoclavicular; CL, Clavicle length; ICC, Intraclass correlation coefficient; ID, Implant distance.

### Assessment of Clinical Results

In terms of clinical outcomes, the mean CMS score at last follow‐up was 89.5 ± 10.5 (median: 94) (range: 63–100) for Group 1 and 89.1 ± 6.1 (median: 90) (range: 76–100) for Group 2 (*p* = 0.325). Preoperative CMS could not be assessed because patients were not suitable for assessment due to post‐traumatic pain. Preoperative and final follow‐up VAS scores were significant for both groups (*p* < 0.01 for both groups). The mean percentage change in VAS score was 71.0 ± 11.6 (median: 71.4) in Group 1 and 77.5 ± 12.1 (median: 80.6) in Group 2 (*p* = 0.102) (Table 4).

**TABLE 4:** Evaluation of clinical results.

Parameters	Total	Open	Arthroscopic	Statistic value	*p*
	n:37 (100%)	n:21 (56.8%)	n:16 (43.2%)		
Last follow‐up CMS^a^	89.3 ± 8.8 [90]	89.5 ± 10.5 [94]	89.1 ± 6.1 [90]	−0.983	0.325^c^
VAS Score^a^					
Preoperative	8.0 ± 1.0 [8]	7.9 ± 1.0 [8]	8.1 ± 1.0 [8]	−0.387	0.699^c^
Last follow‐up	2.1 ± 0.9 [2]	2.2 ± 0.8 [2]	1.8 ± 1.0 [2.5]	−1.601	0.109^c^
*p* ^d^	<0.01	<0.01	<0.01		
Change between preoperative and last final follow‐up (%)	73.8 ± 12.1 [75]	71.0 ± 11.6 [71.4]	77.5 ± 12.1 [80.6]	−1.635	0.102^c^
Complications (n)					
None	24 (64.9%)	12 (57.1%)	12 (75.0%)	1.271	0.315^b^
Yes	13 (35.1%)	9 (42.9%)	4 (25.0%)		
ACJ Arthritis	4 (10.8%)	3 (14.3%)	1 (6.3%)		
Implant Failure	4 (10.8%)	3 (14.3%)	1 (6.3%)		
CC LC	3 (8.1%)	1 (4.8%)	2 (12.5%)		
CTO	2 (5.4%)	2 (9.5%)	0 (0.0%)		

Abbreviations: ACJ, acromioclavicular joint; CC LC, coracoclavicular ligament calcification; CMS, constant Murray score; CTO, clavicular tunnel osteolysis; VAS, visual analog scale.

^a^ Data are presented as means ± standard deviations [median].

^b^ Chi‐square.

^c^ Mann–Whitney U.

^d^ Wilcoxon.

### Reduction Loss and Other Complications

In order to evaluate relationship between reduction loss and last follow‐up CMS with implant position, all patients were divided into three groups according to ID/CL ratios “≤0.169”, “0.17–0.24”, and “≥0.241”. Although not statistically significant, reduction was more maintained in the “0.17–0.24” (acceptable implant position) group with 16 patients (57.1%) compared to the other two groups (*p* = 0.430). Furthermore, the mean CMS at the last follow‐up was 91.8 ± 5.1 (median: 90.5) in the “0.17–0.24” group, which was higher than the other groups (*p* = 0.559) (Table 5).

**TABLE 5:** Comparison of “acceptable implant position” with implant distance‐clavicle length ratio, reduction loss, and constant Murray score.

Parameters	Acceptable implant position			Statistic value	*p*
	≤0.169	0.17–0.24	≥0.241		
	(n:11)	(n:19)	(n:7)		
ID/CL^a^	0.12 ± 0.03 [0.12]	0.21 ± 0.02 [0.21]	0.27 ± 0.01 [0.27]	30.168	<0.001^c^
Reduction Loss					
None (n:28)	7 (25.0%)	16 (57.1%)	5 (17.9%)	1.687	0.430^b^
Yes (n:9)	4 (44.4%)	3 (33.3%)	2 (22.3%)		
Last follow‐up CMS^a^	87.6 ± 11.5 [90]	91.8 ± 5.1 [91]	85.1 ± 10.8 [84]	1.690	0.430^c^

Abbreviations: CMS, constant Murray score; ID/CL, implant distance/clavicle length ratio.

^a^ Data are presented as means ± standard deviations [median].

^b^ Chi‐square.

^c^ Kruskal–Wallis.

Complications were observed in nine (42.9%) patients in Group 1 and four (25.0%) patients in Group 2 (*p* = 0.315). The most common complications in Group 1 were ACJ arthritis (14.3%) and implant failure (14.3%), while in Group 2, the most common complication was CC ligament calcification (12.5%) (Table 4).

## Discussion

### Highlights of this Study

The results of this study showed that, although not statistically significant, less reduction loss and higher CMS were achieved with a single‐bundle SLD performed in the range of 0.17–0.24 (ID/CL ratio), which is considered as an “acceptable implant position”. In addition, similarly high clinical outcomes and pain relief were achieved in patients with type 3 and 5 ACJ dislocations who underwent both arthroscopic and open surgery.

### Single‐Bundle SLD and Other Implant Options in the Treatment of ACJ Dislocation

More than 100 surgical techniques have been described for the surgical treatment of ACJ dislocation.^14^ Due to the use of so many techniques, it can be mentioned that there is no consensus on an appropriate and gold standard treatment for this trauma yet. Developments in implant technologies lead orthopaedic interventions to be more minimally invasive and biological. With adjustable SLDs, it is aimed to maintain joint reduction until the CC ligaments heal by supporting the stabilization of the ACJ in the vertical direction.^15^ Compared to hook plate, SLD has been reported to exhibit lower complications and higher clinical scores due to its minimally invasive method.^16^ Additionally, it was emphasized that similarly excellent results were obtained with suture anchor and SLD, and there was no difference in terms of ACJ stability.^17^ Adjustable SLDs can be performed in various bundle configurations. Özcafer et al. reported that the CMS of patients with type 5 ACJ dislocation treated with single‐bundle SLD was 93.2 after 19 months of follow‐up.^18^ The CMS of 45 patients with Type 3 and higher ACJ dislocation operated with single‐bundle SLD with a mean follow‐up of 4.8 years was 83.7.^19^ On the other hand, double‐bundle SLD provides better vertical stability compared to single‐bundle.^20^ In the current study, a single‐bundle SLD was used, the CMS for all patients was 89.3 after a mean follow‐up of 22.3 months.

### Different Surgical Procedures of SLD in the Treatment of ACJ Dislocation

ACJ dislocations, like many other orthopaedic procedures, can be performed either open or arthroscopically. Previous studies have indicated that both open and arthroscopic approaches yield similar functional and clinical outcomes. However, it has been reported that arthroscopic procedures tend to have longer operation times and higher costs.^8^, ^21^ Faggiani et al. found that patients who underwent mini‐open surgery returned to sport with improved performance and less pain.^22^ Recently, we have favored an arthroscopic approach to ACJ dislocation. In terms of operation time, it is almost shorter than open surgery. The reason for the similar durations of both methods in our results is that the initial cases in the arthroscopic approach took longer due to the learning curve. The arthroscopic approach has become our current preference due to its advantages, including fewer and smaller incisions, more minimally invasive procedures, and better preservation of soft tissue integrity. The similarly high clinical results obtained in this study for both approaches are consistent with the literature.

### Reduction Loss and Other Complications Associated with SLD

The most common postoperative complication of ACJ dislocation is loss of reduction.^23^ The incidence of reduction loss was found to be 24% in patients operated on with the mini‐open technique using a single‐bundle SLD and 25% in patients who underwent arthroscopic surgery.^10^, ^11^ In addition, reduction loss rates were 33.0% and 3.2% in patients operated on with single and double‐bundle SLD, respectively.^9^, ^20^ The definition of “reduction loss” is not clear in the literature. In some studies, the change in the CC distance measured at the last follow‐up in millimeters compared to postoperative day 1 is accepted as “reduction loss”, whereas in some studies, a change of more than 50% in this distance is accepted as “reduction loss”.^9^, ^24^ In our study, “reduction loss” was defined as a change of 50% or more when comparing the CC distance measured at the last follow‐up with the measurement at postoperative day 1. Accordingly, while the overall reduction loss was 24.3% in all patients, it was 19.1% in the open procedure group and 31.3% in the arthroscopic procedure group. Other complications include ACJ arthritis, clavicle fracture, implant failure, and bone erosion.^7^, ^9^ In a meta‐analysis, no difference was found between open and arthroscopic operated ACJ dislocations in terms of reduction loss and other complications.^25^ Consistent with the literature, our study revealed no significant difference between open and arthroscopic operations in terms of reduction loss. Similarly, the incidence of other complications aligned with existing literature findings.

### Effect of Clavicular Tunnel Position on Reduction Loss

Clavicular tunnel position is one of the causes of reduction loss in ACJ dislocations.^26^ Studies investigating the anatomy of the AC joint have reported that the length of the clavicle and the attachment points of the CC ligaments (trapezoid and conoid) to the clavicle vary between individuals, genders, and races.^27^, ^28^ As mentioned above, the study referenced in this paper observed that although the distances of the attachment points of the trapezoid and conoid ligaments to the lateral edge of the clavicle were different, the ratio of this distance to the length of the clavicle was constant. This ratio is 0.17 for the trapezoid ligament and 0.24 for the conoid ligament.^12^ Shibata et al. found the trapezoid ratio to be 0.13 in males and 0.12 in females, and the conoid ratio to be 0.24 and 0.23, respectively.^28^ In patients with ACJ dislocation operated on with double clavicular tunnel using free tendon graft, reduction was maintained with a ratio of 0.128 in trapezoid tunnel and 0.248 in conoid tunnel.^29^ In our study using a single‐bundle SLD, we observed that patients with a tunnel position between 0.17 and 0.24 exhibited the least reduction loss.

### Clinical Outcomes Associated with SLD in the Treatment of ACJ Dislocation

Despite high rates of reduction loss and other complications, studies have demonstrated that patients with ACJ dislocation who underwent surgery with SLD achieved high clinical scores and reported low postoperative pain. Çarkçı et al. reported a mean CMS of 92.03 after a mean follow‐up of 31 months in 36 patients who underwent arthroscopic surgery with a single‐bundle SLD.^10^ Additionally, CMS scores of 92.9 and 94.8 were achieved in patients operated with single‐ and double‐bundle SLD, respectively.^20^ In a study comparing single‐bundle SLD with open and arthroscopic surgery, CMS was found 84.45 and 84.18 at the last follow‐up, respectively.^8^ In the comparison between 66 patients who operated with single and double SLD, the mean VAS scores were 0.4 and 0.3, respectively, at the second year of follow‐up.^30^ Supporting the existing literature, all patients included in our study achieved high clinical scores at final follow‐up. Furthermore, the VAS decreased by 73.8% compared to the preoperative period, with an average score of 2.1. In addition, higher CMS was obtained in patients with an ID/CL ratio between 0.17 and 0.24.

Based on the findings of this study and our clinical practice, positioning the clavicular tunnel between the anatomical attachment points of the CC ligaments on the clavicle is crucial to prevent reduction loss in ACJ patients treated with single‐bundle SLD. Therefore, in preoperative planning, calculating the clavicular tunnel position based on the ID/CL ratio of 0.14–0.21 is recommended. Finally, we attribute the lower complication rate associated with arthroscopic procedures to their minimally invasive nature and preservation of the biological environment.

### Limitations and Prospect

The limitations of our study are the small number of cases, the retrospective study without randomization, the inability to compare with other implants, and the inability to determine the time of reduction loss due to the lack of regular visits to outpatient clinics. In future studies, the factors affecting the results in standardized groups should be investigated.

## Conclusions

Patients with acute type 3 and 5 ACJ dislocations operated with open or arthroscopic procedures with single‐bundle SLD demonstrate high clinical and functional scores despite potential reduction loss challenges. It is suggested that preparing the clavicular tunnel with an ID/CL ratio between 0.17 and 0.24 may contribute to sustaining ACJ reduction in the postoperative period.

## Conflict of Interest Statement

The authors report no conflicts of interest.

## Ethics Statement

This study was performed in line with the principles of the Declaration of Helsinki. Approval was granted by the Ethics Committee of Istanbul Training and Research Hospital. (Date: 04.08.2023 No: 202).

## Author Contributions

All authors contributed to the study. Conceptualization/Methodology: Ahmet Şenel, Ömer Cihan Batur, and Oğuz Kaya. Data Collection: Selman Sert, Oğuz Kaya, and Sefa Key. Statistical analyses: Ahmet Şenel, Murat Eren. Literature research: Murat Eren, Selman Sert, and Ömer Cihan Batur. Writing—Original Draft: Ahmet Şenel. Writing—Review & Editing: Ahmet Şenel, Oğuz Kaya, and Sefa Key. Visualization: Murat Eren, and Selman Sert. Supervision: Ahmet Şenel and Sefa Key. All authors commented on previous versions of the manuscript. All authors read and approved the final manuscript.

## Authorship Declaration

All authors listed meet the authorship criteria according to the latest guidelines of the International Committee of Medical Journal Editors. All authors are in agreement with the manuscript.
